# Verhütung auf YouTube, Instagram und TikTok: Eine Inhalts- und Qualitätsanalyse

**DOI:** 10.1007/s00103-023-03698-0

**Published:** 2023-05-09

**Authors:** Nicola Döring, Stephan Lehmann, Claudia Schumann-Doermer

**Affiliations:** 1 IfMK, TU Ilmenau, Ehrenbergstraße 29, 98693 Ilmenau, Deutschland; 2 Deutsche Gesellschaft für psychosomatische Frauenheilkunde und Geburtshilfe (DGPFG), Dresden, Deutschland

**Keywords:** Gesundheitsinformationen, Verhütungsinformationen, Informationsqualität, Internet, mDISCERN-Index, Health information, Contraceptive information, Information quality, Internet, mDISCERN index

## Abstract

**Hintergrund:**

Jugendliche und Erwachsene beziehen Informationen über Verhütung zunehmend über soziale Medien.

**Ziel der Arbeit:**

Vor diesem Hintergrund ist es Ziel der vorliegenden Studie, erstmals Inhalte und Qualität deutschsprachiger Verhütungsbeiträge auf YouTube, Instagram und TikTok zu untersuchen. Beantwortet werden sollen Forschungsfragen zu Anbieter*innen (F1), Inhalten (F2) und Qualität der Verhütungsbeiträge (F3) sowie zu Publikumsreaktionen (F4).

**Material und Methoden:**

Es wurde eine Stichprobe von *N* = 1000 Verhütungsbeiträgen von YouTube (500), Instagram (250) und TikTok (250) gezogen. Pro Beitrag gingen maximal 20 verhütungsbezogene Kommentare in das Kommentar-Sample ein (*N* = 12.454). Die Beiträge und Kommentare wurden mittels reliabilitätsgeprüfter Codebücher analysiert. Die Datenanalyse erfolgte mit SPSS. Die Studie ist präregistriert und alle Daten, Materialien und Analyseskripte sind öffentlich verfügbar.

**Ergebnisse:**

Es zeigte sich, dass die Verhütungsbeiträge mehrheitlich von Gesundheitslaien stammten (52 %), gefolgt von Medienprofis und Gesundheitsprofis (F1). Inhaltlich deckten die Verhütungsbeiträge alle verfügbaren Verhütungsmethoden ab, wobei Pille (69 %) und Kondom (40 %) dominierten (F2). Nach gängigen Qualitätskriterien zeigten sich deutliche Defizite, wobei im Vergleich YouTube-Videos am besten abschnitten (F3). TikTok-Videos dagegen waren Spitzenreiter bei den Publikumsreaktionen, sie verzeichneten die meisten Views, Likes und Kommentare. Die Kommentarspalten wurden vom Publikum oft genutzt, um eigene Verhütungserfahrungen zu teilen oder Nachfragen zu stellen (F4).

**Diskussion:**

Weitere Forschung sowie Praxismaßnahmen sind notwendig, um die Qualität von Verhütungsinformationen in sozialen Medien besser einschätzen und optimieren zu können.

## Abstract (en)

**Background:**

Young people and adults increasingly obtain information about contraception via social media.

**Research aim:**

Against this background, the aim of this study is to investigate for the first time the content and quality of German-language contraceptive posts on YouTube, Instagram, and TikTok. Research questions on providers (F1), content (F2), and quality of contraceptive posts (F3) as well as audience reactions (F4) will be answered.

**Materials and methods:**

A sample of *N* = 1000 contraceptive posts from YouTube (500), Instagram (250), and TikTok (250) was drawn. A maximum of 20 contraception-related audience comments per post were included in the comment sample (*N* = 12,454). Posts and comments were analyzed using reliability-tested codebooks. SPSS was used for data analysis. The study is preregistered and all data, materials, and analysis scripts are publicly available.

**Results:**

The majority of contraceptive posts were found to be from health laypersons (52%), followed by media professionals and health professionals (F1). In terms of content, contraceptive posts covered all available contraceptive methods, with the pill (69%) and condoms (40%) dominating (F2). According to common quality criteria, there were clear deficits, with YouTube videos scoring best in comparison (F3). TikTok videos, on the other hand, were top performers in terms of audience interaction, attracting the most views, likes, and comments. Comment columns were often used by the audience to share their own contraceptive experiences or ask follow-up questions (F4).

**Discussion:**

Future research as well as practice interventions are needed to further assess the quality of contraceptive information in social media and improve it in a more targeted way.

## Hintergrund

*Verhütung* (engl. „contraception“) umfasst alle Methoden, die absichtlich angewendet werden, um eine Empfängnis bzw. Zeugung durch Geschlechtsverkehr zu verhindern [1]. Es wird heute in der Gesundheitsforschung und Gesundheitsversorgung als *sexuelles und reproduktives Menschenrecht* angesehen, allen Menschen die Möglichkeit zu geben, durch Informationen über und Zugang zu Verhütungsmethoden wie Kondom, Pille, Spirale oder Sterilisation selbst über die eigene Fruchtbarkeit, Fortpflanzung und Familienplanung zu bestimmen [2].

Beim Zugriff auf *Informationen über Verhütungsmethoden* spielt mittlerweile das Internet eine zentrale Rolle: In Deutschland geben Frauen im Alter zwischen 18 und 49 Jahren die gynäkologische Praxis (80 %) und das Internet (29 %) als ihre beiden wichtigsten Informationsquellen zu Verhütungsmethoden an [3]. Für Männer im selben Alter sind das Internet (40 %) sowie Familie und Freunde (40 %) die beiden wichtigsten Informationsquellen zu Verhütungsmethoden [3]. Jugendliche nennen Schulunterricht (69 %), Gespräche (68 %) und das Internet (59 %) als ihre 3 wichtigsten Quellen der Aufklärung über Verhütungsmittel [4].

Wenn *Online-Verhütungsinformationen* so eine große Rolle spielen, stellt sich die Frage, wie sie beschaffen sind. Eine hohe Qualität ist hier den fachlich geprüften Verhütungsinformationen zuzuschreiben, die auf den *Websites *des Berufsverbandes der Frauenärzte e. V.^1^, von pro familia Deutschland^2^ und von der Bundeszentrale für gesundheitliche Aufklärung (BZgA)^3^ bereitgestellt werden. Wer im Internet per Suchmaschine (meist Google) nach einzelnen Verhütungsmethoden sucht, wird mit den ersten Treffern nicht selten auf die genannten Fachportale geleitet, gelangt aber auch oft zu Artikeln der Online-Enzyklopädie Wikipedia. Eine Inhalts- und Qualitätsanalyse der deutschsprachigen *Wikipedia-Beiträge* zu allen verfügbaren Verhütungsmethoden hat große Qualitätsdifferenzen zwischen den Beiträgen und eine eher mittelmäßige Gesamtqualität aufgezeigt, vor allem durch Defizite bei der Vollständigkeit, Aktualität und Verständlichkeit [5].

Doch das Informationsverhalten gerade von jungen Menschen im Netz beschränkt sich nicht darauf, nach interessierenden Verhütungsmethoden zu googeln, also Verhütungsinformationen aktiv abzurufen. Sie bewegen sich täglich auf Social-Media-Plattformen wie YouTube, Instagram und TikTok und bekommen dort auch ohne aktive Suche verhütungsbezogene Beiträge vom Plattformalgorithmus zugespielt und vorgeschlagen [6–8]. Dementsprechend erreichen Verhütungsbeiträge auf solchen Plattformen teilweise enorme Reichweiten. So konnte beispielsweise das TikTok-Video „Wie benutze ich ein Kondom richtig?“ vom Kanal „doktorsex“^4^ binnen eines Jahres rund 1,5 Mio. Views sammeln, während der Kondom-Beitrag in der Wikipedia^5^ binnen eines Jahres rund 200.000-mal und der Kondom-Artikel auf der BZgA-Plattform Familienplanung.de^6^ rund 50.000-mal abgerufen wurde [5].

Vor diesem Hintergrund ist es Ziel der vorliegenden Studie, erstmals systematisch für den deutschsprachigen Raum zu untersuchen, wie und von wem Verhütung auf YouTube, Instagram und TikTok dargestellt wird und wie das Publikum auf die Verhütungsbeiträge reagiert.

## Forschungsstand

Die Verbreitung sexueller Gesundheitsinformationen in sozialen Medien stößt in den letzten Jahren auf wachsendes Forschungsinteresse [9]. Das betrifft auch Online-Verhütungsinformationen: Typischerweise wird mit der Methode der Medieninhaltsanalyse anhand unterschiedlicher Materialstichproben untersucht, wie verschiedene Verhütungsmethoden auf unterschiedlichen Social-Media-Plattformen repräsentiert sind: Untersucht wurden beispielweise Online-Diskussionen über hormonelle Verhütung für den Mann [10] sowie über den Coitus interruptus [11, 12] auf der Forenplattform *Reddit*. Weiterhin wurden verhütungsbezogene Online-Diskussionen auf der Microblogging-Plattform *Twitter *analysiert, und zwar über die Verhütungsspritze [13], das Verhütungsimplantat [14] und die Notfallverhütung [15]. Auch journalistische Twitter-Beiträge über Frauengesundheit und Verhütung waren Gegenstand einer Studie [16]. Ebenso wurden auf der Messenger-Plattform *Snapchat* [17] Verhütungsinformationen betrachtet.

Für die in Deutschland unter Jugendlichen und jungen Erwachsenen besonders populären Social-Media-Plattformen YouTube, Instagram und TikTok liegen ebenfalls erste Studien vor. So wurden die Darstellung der männlichen Sterilisation [18], des Hormonimplantats [19] und der Spirale [20] auf der Video-Plattform *YouTube* inhaltsanalytisch ausgewertet, einschließlich YouTube-Videos zur Selbstentfernung von Implantaten und Spiralen [21]. Auf der Foto- und Video-Plattform *Instagram* [22] wurde Werbung für natürliche Familienplanung erforscht. Auf der Kurzvideoplattform *TikTok *wurden Verhütungsinformationen allgemein [23] sowie die Darstellung der Spirale [24] untersucht.

In der Gesamtschau zeigt sich, dass bisherige Studien zwar den Austausch von Verhütungsinformationen auf Social-Media-Plattformen als prinzipiell nützlich würdigen, aber zahlreiche Qualitätsdefizite dieser Informationen (z. B. Unvollständigkeit, faktische Fehler) monieren sowie auf Verzerrungen in den Online-Diskursen (z. B. Übergewicht an negativen Erfahrungsberichten) hinweisen.

## Forschungsziel

Da sich die bisherige Forschung vor allem auf englischsprachiges Material bezieht und meist einzelne Methoden auf einzelnen Plattformen in den Blick nimmt, zielt die vorliegende Untersuchung darauf ab, auf der Basis eigener Vorstudien [6–8] erstmals einen breiten quantitativen Überblick über deutschsprachige Verhütungsinformationen in sozialen Medien zu geben und dabei die 3 führenden Plattformen YouTube, Instagram und TikTok vergleichend einzubeziehen. Dementsprechend sind folgende Forschungsfragen zu beantworten:

### F1.

Wer bietet auf YouTube, Instagram und TikTok Informationsbeiträge über Verhütung an?

### F2.

Welche Inhalte haben diese Informationsbeiträge?

### F3.

Welche Qualität haben diese Informationsbeiträge?

### F4.

Welche Publikumsreaktionen (Views, Likes, Kommentare) zeigen sich bei diesen Informationsbeiträgen?

## Methode

### Untersuchungsdesign

Zur Beantwortung der 4 Forschungsfragen wurde im Jahr 2022 eine Inhalts- und Qualitätsanalyse als quantitative Querschnittstudie durchgeführt. Die Studie ist präregistriert und folgt der Open-Science-Bewegung, das heißt, die Präregistrierung, das Messinstrument, die Datensätze, das Auswertungsskript sowie zusätzliche Ergebnistabellen sind auf dem Server der Open-Science-Foundation hinterlegt (https://osf.io/tsnuq). Das Untersuchungsmaterial besteht aus öffentlich zugänglichen Social-Media-Beiträgen und -Kommentaren, die nach aktuellem Verständnis der Online-Forschungsethik für wissenschaftliche Untersuchungen frei zur Verfügung stehen [25]. Stichprobe, Datenerhebung und Datenanalyse werden im Folgenden erläutert.

### Stichprobenbildung

Die Studie basiert auf einer Stichprobe von *N* = 1000 Verhütungsbeiträgen von YouTube, Instagram und TikTok und *N* = 12.454 zugehörigen Publikumskommentaren. Die Verhütungsbeiträge wurden über die Suche nach „Verhütung“ sowie nach einzelnen Verhütungsmethoden [5] identifiziert. Es wurden die Top-Beiträge aus den jeweiligen Plattform-Rankings ausgewählt. Eingeschlossen wurden nur deutschsprachige Beiträge, die Verhütung als zentrales Thema sachlich behandeln. Zu jedem Beitrag wurden die 20 meistgelikten verhütungsbezogenen Publikumskommentare erhoben. Da nicht zu allen Beiträgen 20 verhütungsbezogene Kommentare existierten (sondern im Mittel 12), ergab sich ein Sample, das kleiner ist als das theoretische Maximalsample von 20.000 Kommentaren. Ausgeschlossen wurden Publikationskommentare ohne Verhütungsbezug (z. B. Grüße, Witze, Werbung). Die Zusammensetzung der Stichproben ist Tab. 1 zu entnehmen. Die Entscheidung, jeweils Top-Beiträge und Top-Kommentare auszuwählen, basiert auf der Überlegung, dass diese Beiträge und Kommentare die höchste Wahrscheinlichkeit haben, vom Publikum wahrgenommen zu werden.

**Tab. 1:** Zusammensetzung der Stichprobe verhütungsbezogener Beiträge und Kommentare

	YouTube	Instagram	TikTok
Anzahl der Beiträge (*N* = 1000)	500	250	250
Suchbegriff(e)	*Verhütung, Verhütungsmittel, Verhütungsmethode* 25 Verhütungsmethoden^a^	*Verhütung*^b^	*Verhütung*^b^
Auswahl der Beiträge	Auswahl nach Reihenfolge der von YouTube präsentierten deutschsprachigen verhütungsbezogenen Suchergebnisse nach Anzahl der Aufrufe (absteigende Reihenfolge)	Auswahl nach Reihenfolge der von Instagram präsentierten deutschsprachigen verhütungsbezogenen Suchergebnisse nach „Top“-Sortierung	Auswahl nach Reihenfolge der von TikTok präsentierten deutschsprachigen verhütungsbezogenen Suchergebnisse^c^
Anzahl der Kommentare (*N* = 12.454)	5653	3238	3563
Auswahl der Kommentare	Top 20 meistgelikte Kommentare mit Verhütungsbezug pro ausgewählter Beitrag	Top 20 meistgelikte Kommentare mit Verhütungsbezug pro ausgewählter Beitrag	Top 20 meistgelikte Kommentare mit Verhütungsbezug pro ausgewählter Beitrag

^a^ Folgende 25 Verhütungsmethoden wurden in die Suche eingeschlossen [5]: Pille, Coitus interruptus, Diaphragma, Dreimonatsspritze/Hormonspritze/Depotspritze, Femidom/Frauenkondom, Hormonpflaster/Verhütungspflaster, Implanon/Hormonstäbchen/Hormonimplantat, Kondom, Kupferkette, Kupferball, Kupferspirale, Hormonspirale, Knaus-Ogino-Methode/Kalendermethode, Temperaturmethode, symptothermale Methode/natürliche Familienplanung, LAM-Methode (Laktationsamenorrhö-Methode), Minipille, Pille danach, Pille für den Mann, Portiokappe, Spermizid, Sterilisation, Vasektomie, Vaginalring/Hormonring/Verhütungsring, Verhütungsschwamm und Zykluscomputer

^b^Durch eine Vorrecherche wurde festgestellt, dass dieser Suchbegriff ausreichend ist

^c^Die TikTok-Desktop-Version bietet, anders als die mobile App, zum Zeitpunkt der Erhebung keine Filtermöglichkeit. Die Top 250 Videos der Desktop-Suche wurden mit den Ergebnissen der App-Suche verglichen, die sich neben dem Veröffentlichungsdatum (Filterung von „gestern“ bis „letzte 6 Monate“) zudem in „Relevanz“ oder „Anzahl der Likes“ sortieren lassen. Alle Sortierungen stimmten im hohen Maße mit den Ergebnissen der Desktop-Variante überein

### Instrument

Zur Beantwortung der Forschungsfragen wurden je ein Codebuch für die Verhütungsbeiträge der 3 Plattformen sowie ein Codebuch für die Kommentare entwickelt, teils induktiv anhand des Materials, teils deduktiv anhand der Fachliteratur. Die Codebücher für die Verhütungsbeiträge auf YouTube, Instagram und TikTok nehmen vereinzelt auf Plattformspezifika Bezug, folgen ansonsten aber demselben Schema und gliedern sich in 5 Blöcke:

1. *Formale Variablen*: Sie erfassen allgemeine Merkmale der Beiträge (z. B. Link zum Beitrag, Titel des Beitrags, Veröffentlichungsdatum, Videolänge)
2. *Typ des Informationsanbieters*: Zur Beantwortung von F1 wurden verschiedene Typen von Informationsanbietern differenziert. Besonders wichtig war hier gemäß der Fachliteratur zur Online-Gesundheitskommunikation (z. B. [26, 27]), ob Verhütungsbeiträge von Gesundheitsprofis stammen (z. B. von medizinischem Fachpersonal) oder von Gesundheitslaien (z. B. von Anwender*innen einer Verhütungsmethode). Weitere wichtige Anbietertypen sind Medienprofis (z. B. Nachrichtensender) und Unternehmen (z. B. Kondomhersteller). Sofern es sich beim Informationsanbieter um eine natürliche Person handelte, wurde das Geschlecht erfasst.
3. *Verhütungsinhalte*: Zur Beantwortung von F2 wurde in Anlehnung an die Fachliteratur zur Online-Verhütungskommunikation [5] codiert, 1) welche Verhütungsmethode(n) der Beitrag behandelt, 2) ob er Faktenwissen und/oder Erfahrungswissen vermittelt und 3) ob er die Verhütungsmethoden neutral, positiv, negativ oder ambivalent darstellt.
4. *Inhaltsqualität*: Zur Beantwortung von F3 wurden die Beiträge mit dem etablierten Messinstrument zur Qualität von Online-Gesundheitsinformationen beurteilt, dem DISCERN-Index von Charnock et al. [28]. In der vorliegenden Analyse wurde der modifizierte DISCERN-Index (mDISCERN) für soziale Medien verwendet [29]. Der mDISCERN beinhaltet 5 Qualitätskriterien: 1. die Nennung der Ziele eines Beitrags, 2. die Verwendung von zuverlässigen Informationsquellen, 3. die ausgewogene und unvoreingenommene Informationsdarstellung, 4. die Angabe von weiterführenden Informationen und 5. die Nennung von Kontroversen oder Unsicherheiten. Die Beurteilung der Einzelkriterien fließt in einen mDISCERN-Gesamtwert ein mit einem Wertebereich von 0 (schlechteste Qualität) bis 5 (beste Qualität). Zusätzlich wurde anhand der Fachliteratur zur Online-Verhütungskommunikation [5] erfasst, wie vollständig die Verhütungsmethoden dargestellt werden, d. h. inwiefern die 7 Hauptaspekte, die für jede Verhütungsmethode relevant sind (1. Wirkungsmechanismus, 2. Sicherheit, 3. Anwendung, 4. Vorteile, 5. Nachteile, 6. Kosten und 7. Gegenanzeige), behandelt werden. Schließlich wurde die fachliche Korrektheit bzw. das Auftreten von Fehlinformationen codiert. Die Korrektheit der in den Beiträgen beinhalteten Verhütungsinformationen wurde anhand aktueller gynäkologischer Fachliteratur [30–32] überprüft. Zudem wurde auf die Expertise einer auf Verhütung spezialisierten gynäkologischen Fachärztin (Autorin 3) zurückgegriffen.
5. *Quantitative Publikumsreaktionen*: Zur Beantwortung von F4 wurden für jeden Beitrag 1) die Anzahl der Views, 2) die Anzahl der Likes und 3) die Anzahl der Kommentare erfasst. Diese Social-Media-Metriken werden unter dem jeweiligen Social-Media-Beitrag angegeben und zeigen, wie stark das Publikum mit dem Beitrag interagiert.

Das Codebuch für die Kommentare gliedert sich in 2 Blöcke.

1. *Formale Variablen:* Sie erfassen, auf welchen Beitrag sich der Kommentar bezieht, und dokumentieren den Wortlaut des Kommentars.
2. *Qualitative Publikumsreaktionen: *Zur Beantwortung von F4 bezüglich der Publikumsreaktionen wurden neben den oben genannten Social-Media-Metriken als qualitative Indikatoren auf der Basis der Fachliteratur zur Online-Verhütungskommunikation [6–8] auch die Inhalte der verhütungsbezogenen Kommentare codiert: 1. verhütungsbezogene Nachfrage, 2. verhütungsbezogene Zusatzinformation, 3. eigene Erfahrung mit Verhütungsmethode(n), 4. Zustimmung zum Inhalt des Beitrags sowie 5. Bewertung der behandelten Verhütungsmethode (neutral, positiv, negativ, ambivalent).

Die Reliabilität der Codebücher für die Beiträge wurde anhand von 150 zufällig ausgewählten Beiträgen aus dem Sample durch 5 geschulte unabhängige Codierende erfasst. Berechnet wurde der im Feld der Medieninhaltsforschung etablierte Reliabilitätskoeffizient Krippendorffs Alpha für alle Variablen im Codebuch. Für die 3 Beitragscodebücher ergaben sich folgende mittlere Reliabilitätswerte: 0,85 für YouTube, 0,89 für Instagram und 0,86 für TikTok, was jeweils einer guten Messgenauigkeit entspricht. Die Reliabilität der Variablen im Codebuch für die Kommentare wurde anhand von 1000 Kommentaren durch 2 Codierende erfasst und ergab einen mittleren Reliabilitätswert von 0,86, was ebenfalls auf gute Messgenauigkeit hinweist [33].

### Datenerhebung und Datenanalyse

Die Datenerhebung erfolgte im Rahmen manueller Codierung durch 5 geschulte Codierende. Grundlage der Codierung waren dabei die oben dargestellten 4 reliabilitätsgeprüften Codebücher. Die Datenanalyse erfolgte deskriptiv- und inferenzstatistisch (Häufigkeitsanalysen, Chi-Quadrat-Tests bzw. Fishers exakte Tests und Varianzanalysen) unter Nutzung der Software SPSS (Version 26).

## Ergebnisse

### Anbieter von Verhütungsbeiträgen in sozialen Medien

Die Darstellung von Verhütung in sozialen Medien wird quantitativ von *Gesundheitslaien *dominiert: Gut die Hälfte der Top-Verhütungsbeiträge im Sample stammen von Gesundheitslaien (Tab. 2). Dazu gehört beispielsweise das YouTube-Video „Warum ich die Pille nicht mehr nehme“ von „Dagi Bee“^7^, die mit rund 4 Mio. Abonnent*innen eine der reichweitenstärksten YouTube-Influencer*innen Deutschlands ist. Auf der Kurzvideoplattform TikTok ist der Anteil der Top-Verhütungsbeiträge von Gesundheitslaien mit 60 % am höchsten. Hier nimmt beispielsweise die 22-jährige „Lilielouiselias“ aus Berlin (gut 40.000 Follower*innen) das Publikum mit durch den Tag, an dem sie sich eine Kupferspirale einsetzen lässt^8^.

**Tab. 2:** Anbieter*innen der Verhütungsbeiträge (in Prozent der Beiträge im Sample)

Anbietertypen	Gesamt *N* = 1000	YouTube *n* = 500	Instagram *n* = 250	TikTok *n* = 250	*p*
Gesundheitslaien	52	49	47	60	0,004
Medienprofis	18	18	26	8	< 0,001
Gesundheitsprofis	17	16	10	28	< 0,001
Unternehmen^a^	10	13	9	3	< 0,001
Sonstige^a^	4	3	8	1	< 0,001

Prozentwerte basierend auf Top-Verhütungsbeiträgen in absteigender Reihenfolge. Zeilenweise Auswertung mit zweidimensionalen Chi-Quadrat-Tests. ^a^Aufgrund niedriger Zellenbesetzungen wurde Fishers exakter Test gerechnet

Auch ausgebildete *Medienprofis* tragen zur Verhütungskommunikation in sozialen Medien bei, etwa das ARD/ZDF-Content-Netzwerk „funk“ mit seinen Formaten „Auf Klo“ (vertreten auf YouTube und Instagram) und „mädelsabende“ (vertreten auf Instagram), die immer wieder Verhütungsfragen aufgreifen. In Instagram-Beiträgen von „mädelsabende“ wird beispielsweise die Sterilisation als Verhütungsmethode für Männer^9^ ebenso wie für Frauen^10^ thematisiert und dabei auf der Basis von Rechercheergebnissen auf die weltweite Verbreitung der letztgenannten Methode hingewiesen.

Rund jeder fünfte Top-Verhütungsbeitrag in den betrachteten sozialen Medien stammt von ausgebildeten *Gesundheitsprofis*. Ein typischer Vertreter dieses Anbietertyps ist der TikTok-Kanal „doktorsex“, der von der Gynäkologin Dr. Sheila de Liz zusammen mit dem Urologen Volker Wittkamp betrieben wird und neben anderen Themen der Sexualaufklärung auch diverse Verhütungsbeiträge anbietet. So demonstriert beispielsweise die Gynäkologin in einem 22 s langen TikTok-Video^11^ anhand eines Gebärmuttermodells das Einsetzen der Spirale.

Nicht zuletzt beteiligen sich Unternehmen am Verhütungsdiskurs in sozialen Medien (Tab. 2), etwa Kondomhersteller oder Anbieter von Zykluscomputern, die dadurch Marketing für ihre Produkte betreiben.

Sofern es sich bei den Informationsanbietenden um eine natürliche Person handelt (und nicht z. B. um ein Unternehmen), sind Frauen in der Überzahl^12^: Der Anteil der Verhütungsbeiträge von Frauen war auf Instagram am größten (98 % Frauen, 2 % Männer), gefolgt von TikTok (84 % Frauen, 16 % Männer) und YouTube (81 % Frauen, 19 % Männer).

### Inhalte von Verhütungsbeiträgen in sozialen Medien

Betrachtet man die Inhalte der Verhütungsbeiträge in sozialen Medien mit Blick auf die repräsentierten Methoden, so zeigt sich, dass viele Beiträge mehrere Verhütungsmethoden vergleichend einbeziehen: Die untersuchten YouTube-Videos behandelten im Mittel 4, die Instagram-Posts und TikTok-Videos im Durchschnitt 2 verschiedene Verhütungsmethoden. Dominiert werden die deutschsprachigen Top-Verhütungsbeiträge in sozialen Medien durch die Pille: Sie wird in 59 % aller Beiträge behandelt (Tab. 3). Auch Kondom, Kupfer- und Hormonspirale sowie Methoden der natürlichen Familienplanung (NFP), Kupferkette und Verhütungsring sind in mindestens jedem zehnten Top-Verhütungsbeitrag vertreten.

**Tab. 3:** Inhalte der Verhütungsbeiträge: behandelte Verhütungsmethoden (in Prozent der Beiträge im Sample)

Verhütungsmethoden		Gesamt *N* = 1000	YouTube *n* = 500	Instagram *n* = 250	TikTok *n* = 250	*p*
1	Pille	59	64	63	46	< 0,001
2	Kondom	40	53	26	29	< 0,001
3	Kupferspirale	27	38	14	17	< 0,001
4	Hormonspirale	19	26	12	13	< 0,001
5	Methoden der NFP^ab^	16	22	15	6	< 0,001
6	Kupferkette^a^	13	21	4	4	< 0,001
7	Verhütungsring^a^	10	16	3	6	< 0,001
8	Diaphragma^a^	9	13	5	5	< 0,001
9	Zykluscomputer	9	8	10	7	0,352
10	Vasektomie^a^	8	11	6	4	0,001
11	Hormonimplantat^a^	7	12	3	2	< 0,001
12	Pille danach^a^	7	13	< 1	3	< 0,001
13	Pille für den Mann^a^	6	6	7	6	0,674
14	Dreimonatsspritze^a^	6	9	2	3	< 0,001
15	Minipille^a^	6	9	2	3	< 0,001
16	Kupferball^a^	6	10	1	2	< 0,001
17	Hormonpflaster^a^	5	8	2	3	< 0,001
18	Coitus interruptus^a^	5	6	2	5	0,011
19	Sterilisation der Frau^a^	4	5	4	1	0,024
20	Femidom^a^	3	4	1	2	0,049
21	Verhütungs-Apps^a^	3	4	2	–	< 0,001
22	Spermizid^a^	2	3	< 1	1	0,041
23	Portiokappe^a^	2	2	< 1	< 1	0,078
24	LAM-Methode^ac^	< 1	1	2	–	0,151
25	Verhütungsschwamm	< 1	< 1	–	–	–

Prozentwerte basierend auf Top-Verhütungsbeiträgen in absteigender Reihenfolge. Die Summe aller Prozentwerte pro Spalte liegt über 100 %, da Beiträge mehrere Verhütungsmethoden behandeln können. Zeilenweise Auswertung mit zweidimensionalen Chi-Quadrat-Tests. ^a^Aufgrund niedriger Zellenbesetzungen wurde Fishers exakter Test gerechnet

^b^*Methoden der NFP* (natürliche Familienplanung) beinhalten Nennungen NFP allgemein, symptothermale Methode, Temperaturmethode und Kalendermethode

^c^*LAM-Methode* (Laktationsamenorrhö-Methode), Methode der natürlichen Empfängnisverhütung durch Stillen

Die meisten Verhütungsbeiträge (54 %) präsentieren reines Faktenwissen, 23 % bringen Fakten- und Erfahrungswissen und 24 % reines Erfahrungswissen. Die in den Beiträgen zu findenden Aussagen über Verhütungsmethoden sind überwiegend neutral (61 %), zu einem nennenswerten Teil aber auch wertend: 21 % der Aussagen über Verhütungsmethoden sind negativ, 14 % positiv und 4 % ambivalent. Der größte Anteil negativer Bewertungen zu Verhütungsmethoden ist auf Instagram zu finden (27 %), dabei wird vor allem die Pille wegen ihrer negativen Nebenwirkungen kritisiert.

### Qualität von Verhütungsbeiträgen in sozialen Medien

**Abb. 1:**
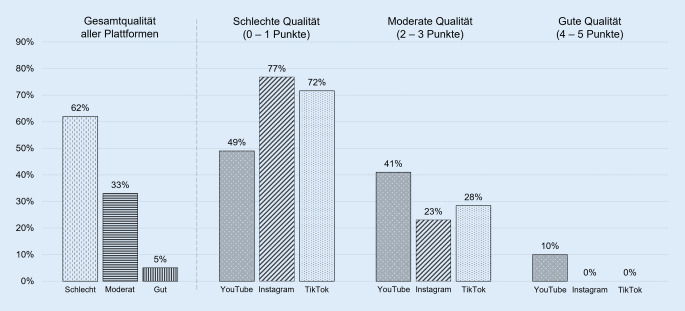
Qualität der Verhütungsinformationen in sozialen Medien (gemessen mit dem mDISCERN-Index, Basis: insgesamt 1000 Top-Verhütungsbeiträge). (Quelle: eigene Abbildung)

Gemessen mit dem mDISCERN-Index haben die Top-Verhütungsbeiträge in sozialen Medien mehrheitlich eine schlechte Informationsqualität bzw. *Zuverlässigkeit* (Abb. 1). Dabei zeigen sich deutliche Differenzen zwischen den Plattformen (zweidimensionaler Chi-Quadrat-Test: *p* < 0,001): YouTube übertrifft in der Inhaltsqualität Instagram und TikTok, da YouTube-Beiträge gemäß den Kriterien des mDISCERN-Index häufiger auf zuverlässige Quellen verweisen, weiterführende Informationen angeben und eine ausgewogene Darstellung der Verhütungsmethoden anbieten. Beispiele für YouTube-Verhütungsvideos mit hoher Informationsqualität (mDISCERN-Wert 5) sind die gut recherchierten und mit vielen Quellen versehenen Videos „Die 10 SICHERSTEN Verhütungsmittel“ von „fraeuleinchaos“^13^ (Anbietertyp: Gesundheitslaie) und „Die Pille wissenschaftlich geprüft“ von „mailab“^14^ (Anbietertyp: Medienprofi).

Bewertet man die präsentierten Verhütungsinformationen hinsichtlich ihrer *sachlichen Korrektheit*, so zeigt sich, dass die Mehrheit der untersuchten Beiträge fehlerfrei ist (85 %). Das gilt für YouTube (86 %), Instagram (84 %) und TikTok (83 %). Dabei ist jedoch zu beachten, dass die Social-Media-Beiträge oftmals kurze und allgemeine Aussagen (z. B. „die Pille ist sicher“, „die Pille ist nicht zu 100 % sicher“) enthalten, die aufgrund ihrer Allgemeinheit kaum als faktisch falsch einzuordnen sind. Faktenfehler lassen sich nur dann feststellen, wenn auch konkrete Fakten genannt werden (z. B. der exakte Pearl-Index). Ein solcher Präzisionsgrad wird von Social-Media-Beiträgen aber nur selten erreicht. Wenn Faktenfehler vorkamen, waren das beispielsweise falsche Angaben über die Sicherheit von Methoden (z. B. Zykluscomputer Daysy wird als „mit die sicherste Verhütungsmethode“ darstellt^15^) oder falsche Angaben über die Anwendung von Methoden (z. B. Darstellung der Vaginaldusche als Verhütungsmittel^16^).

Ein weiteres Maß für die Qualität von Verhütungsinformationen ist die *Vollständigkeit*. So informiert ein Social-Media-Beitrag dann umfassend über eine Verhütungsmethode, wenn er alle 7 Hauptaspekte behandelt (Wirkungsmechanismus, Sicherheit, Anwendung, Vorteile, Nachteile, Kosten, Gegenanzeige). Hier zeigt sich, dass viele Social-Media-Beiträge nur einzelne Aspekte aufgreifen und keine Gesamtinformation vermitteln, wobei der meistbehandelte Aspekt die Nachteile sind: So geht fast jeder zweite (48 %) der Top-Verhütungsbeiträge auf Nachteile ein, aber nicht einmal jeder dritte auf die Sicherheit oder den Wirkungsmechanismus der jeweiligen Verhütungsmethode (Tab. 4).

**Tab. 4:** Repräsentation der Hauptaspekte von Verhütungsmethoden (in Prozent der Beiträge im Sample)

Hauptaspekte von Verhütungsmethoden	Gesamt *N* = 1000	YouTube *n* = 500	Instagram *n* = 250	TikTok *n* = 250	*p*
Nachteile	48	57	48	29	< 0,001
Anwendung	44	62	16	36	< 0,001
Vorteile	38	52	30	19	< 0,001
Sicherheit	28	35	19	21	< 0,001
Wirkungsmechanismus	27	39	15	16	< 0,001
Kosten	15	21	11	6	< 0,001
Gegenanzeige^a^	9	15	3	4	< 0,001

Prozentwerte basierend auf Top-Verhütungsbeiträgen in absteigender Reihenfolge. Zeilenweise Auswertung mit zweidimensionalen Chi-Quadrat-Tests

^a^Aufgrund niedriger Zellenbesetzungen wurde Fishers exakter Test gerechnet

Nachteile dominieren insbesondere den Verhütungsdiskurs auf Instagram, der sich vor allem um die Nachteile der Pille dreht und oft ein „pillenfreies“ oder gar „hormonfreies“ Leben als gesünder und lustvoller propagiert. Auf YouTube und TikTok sind die Aspekte der Anwendung von Verhütungsmethoden etwas stärker präsent als die Nachteile, so findet man etwa YouTube- und TikTok-Videos zur Anwendung des Kondoms oder zum Einsetzen der Spirale.

Aufgrund ihres Umfangs (durchschnittliche Länge M = 10:24 min; SD = 8:11) übertreffen YouTube-Videos hinsichtlich des Informationsgehalts sowohl TikTok-Videos (durchschnittliche Länge M = 0:33 min; SD = 0:26) als auch Instagram-Posts (meist Fotobeiträge und keine Videos), was sich darin widerspiegelt, dass die Hauptaspekte von Verhütungsmethoden in YouTube-Videos vergleichsweise vollständiger vertreten sind (Tab. 4).

### Publikumsreaktionen zu Verhütungsbeiträgen in sozialen Medien

**Abb. 2:**
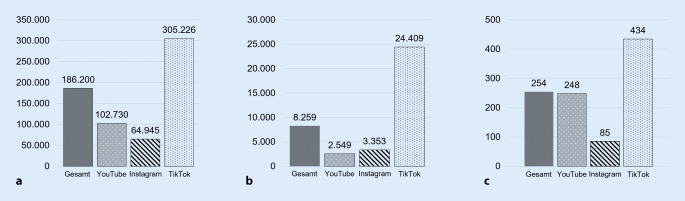
Publikumsreaktionen auf Top-Verhütungsbeiträge in sozialen Medien (Mittelwerte; Basis: insgesamt 1000 Top-Verhütungsbeiträge und deren Social-Media-Metriken). **a** Durchschnittliche *Views* pro verhütungsbezogenem Video, **b** durchschnittliche *Likes* pro verhütungsbezogenem Video, **c** durchschnittliche Anzahl von *Kommentaren* pro Beitrag. (Quelle: eigene Abbildung)

Betrachtet man die Publikumsreaktionen auf die Verhütungsbeiträge, so hebt sich die Kurzvideoplattform TikTok signifikant von YouTube und Instagram ab: Die TikTok-Videos verzeichnen gemäß einfaktoriellen Varianzanalysen (*p* < 0,001) jeweils um ein Vielfaches mehr Views, Likes und Kommentare als YouTube-Videos oder Instagram-Posts (Abb. 2).

Die untersuchten *N* = 1000 Top-Verhütungsbeiträge hatten in Summe (248 × 465^17^) + (85 × 250) + (434 × 250) = 245.070 Kommentare gesammelt, was 254 Kommentaren im Durchschnitt^18^ entspricht (Abb. 2c). Aus diesen wurden jeweils die 20 meistgelikten verhütungsbezogenen Kommentare pro Beitrag in das Kommentar-Sample aufgenommen (siehe Abschnitt Stichprobenbildung). Die Inhaltsanalyse dieser *N* = 12.454 verhütungsbezogenen Kommentare zeigte, dass der Verhütungsdiskurs in den Kommentaren vor allem durch 4 Arten von Kommentarinhalten geprägt ist:

- *Verhütungserfahrungen des Publikums* (38 % der Kommentare): Die Kommentarspalten eröffnen einen Kommunikationsraum für eigene Verhütungserfahrungen. In den Kommentaren zu Beiträgen über die Pille teilen Frauen beispielsweise Erfahrungen mit negativen Nebenwirkungen (z. B. „diese Stimmungsschwankungen … das war bei mir manchmal sehr extrem … auch der Verlust der Libido“), vereinzelt werden positive Erfahrungen geteilt (z. B. „Ich nehme jetzt seit ca. 6–7 Jahren die Pille und habe keine Nebenwirkungen“). Beiträge über die Spirale werden oft kommentiert, indem Frauen eigene Schmerzerfahrungen beim Einsetzen ansprechen (z. B. „Das war so traumatisch das Einsetzen der Spirale … NIE WIEDER! Ich hatte so schlimme Schmerzen, dass ich nicht mehr laufen konnte …“), aber auch Positives mitteilen (z. B. „Vor 3 Tagen ohne Betäubung und Narkose einsetzen lassen. Kurz unangenehm, aber wirklich auszuhalten“).
- *Nachfragen des Publikums* (19 %): Nicht selten nutzt das Publikum die Kommentarspalten zudem, um Rückfragen zu stellen. Wenn ein Video beispielsweise die Anwendung des Kondoms erklärt, wird gefragt: „Wie erkennt man, ob’s richtig rum ist?“ Und zu einem Aufklärungsvideo über den Verhütungsring wird gefragt: „Doofe Frage, aber wie kriegt man den wieder raus?“ oder „Was passiert, falls er rausfällt? Kann das passieren? Beim Kuscheln?“
- *Zusatzinformationen des Publikums* (15 %): Zuweilen ergänzt das Publikum die im Verhütungsbeitrag gegebenen Informationen und benennt beispielsweise Kosten einer Methode oder verweist auf Beratungsstellen.
- *Beurteilung des Verhütungsbeitrags* (7 %): Vergleichsweise selten finden sich in den Top-Kommentaren Beurteilungen des Verhütungsbeitrags, etwa im Sinne von Lob (z. B. „sehr informativer Überblick“) oder Kritik (z. B. „Ich bin fassungslos, wie im Hinblick auf die Pillendiskussion man das Medikament so fahrlässig verharmlosen kann“).

Wenn das Publikum Verhütungsmethoden kommentiert, dann oft neutral (53 %), zu einem nennenswerten Teil aber auch negativ (27 %) und seltener positiv (18 %) oder ambivalent (3 %).

## Diskussion

### Interpretation der Befunde

Es zeigte sich, dass die Top-Verhütungsbeiträge auf den führenden Social-Media-Plattformen YouTube, Instagram und TikTok oft von Gesundheitslaien stammen (F1), was der Intention sozialer Medien als „Mitmach-Medien“ entspricht. Einzelne Medienprofis und Gesundheitsprofis bestimmen den deutschsprachigen Verhütungsdiskurs in sozialen Medien durch reichweitenstarke Beiträge maßgeblich mit, etwa die von Journalist*innen geführten Kanäle „mädelsabende“ (Instagram) und „Auf Klo“ (YouTube, Instagram) des ARD/ZDF-Content-Netzwerks „funk“ und die von Ärzt*innen geführten Kanäle „doktorsex“ (TikTok) und „gynäko.logisch“ (YouTube, Instagram; [7, 8]).

Hinsichtlich der behandelten Inhalte ist erkennbar, dass alle verfügbaren Verhütungsmethoden aufgegriffen werden, wobei Pille, Kondom und Spirale als die meistgenutzten Methoden [3] besonders oft thematisiert werden (F2). Im Einklang mit dem bisherigen Forschungsstand, der Qualitätsdefizite bei Online-Gesundheitsinformationen problematisiert [9], zeigten sich auch bei der hier vorgelegten Qualitätsanalyse deutliche Defizite, etwa bei der Informationsqualität gemäß mDISCERN-Index und der Betrachtung der Vollständigkeit der Informationen, wobei YouTube-Videos die vergleichsweise beste Qualität bieten (F3).

Hinsichtlich der Publikumsreaktionen liegt TikTok vorne: Hier werden die meisten Views, Likes und Kommentare zu Verhütungsbeiträgen verzeichnet (F4). Dabei nutzt das Publikum die Kommentarspalten themenbezogen vor allem, um eigene Verhütungserfahrungen zu teilen und Nachfragen zu stellen. Im Einklang mit anderen Feldern der Gesundheitskommunikation [27] zeigt sich auch beim Verhütungsdiskurs in sozialen Medien ein Bias in Richtung der stärkeren Betonung von negativen Aspekten sowohl in den Beiträgen als auch in den Kommentaren.

### Limitationen

Soziale Medien und ihre Inhalte sind definitionsgemäß sehr dynamisch. Die vorliegende Studie ist dementsprechend eine Momentaufnahme. Ein regelmäßiges Monitoring wäre notwendig, um den aktuellen Stand der Verhütungskommunikation im Zeitverlauf mitzuverfolgen. Obwohl bereits ein relativ großes Sample an deutschsprachigen Verhütungsbeiträgen und zugehörigen Kommentaren von 3 verschiedenen Plattformen untersucht wurde, könnten in zukünftigen Studien noch weitere soziale Medien einbezogen werden (z. B. Facebook, Twitter, Twitch).

### Fazit

Die vorliegende Inhalts- und Qualitätsanalyse beschreibt die Verhütungskommunikation auf YouTube, Instagram und TikTok. Dabei bietet YouTube im Plattformenvergleich die gehaltvollsten Beiträge und auch die längsten Kommentare, hier werden am ehesten verschiedene Methoden verglichen und diverse Aspekte einer Verhütungsmethode einbezogen. Auf Instagram ist die Verhütungskommunikation am stärksten durch den Erfahrungsaustausch junger Frauen über negative Pillennebenwirkungen geprägt. Auf TikTok bekommt ein sehr junges Publikum knappe Verhütungsbeiträge vom Algorithmus zugespielt, was oftmals zu Nachfragen führt. Für die professionelle Sexualaufklärung ergeben sich die beiden Anforderungen, a) mit qualitätsvollem eigenen Content präsent zu sein sowie b) durch zeitgemäße sexualbezogene Medienbildung die Social-Media-Nutzenden zu befähigen, mit verhütungsbezogenen Beiträgen und Kommentaren zielgerichtet und kritisch umzugehen.

## Footnotes

1 www.frauenaerzte-im-netz.de/familienplanung-verhuetung/ (letzter Zugriff: 28.03.2023).

2 www.profamilia.de/themen/verhuetung/ (letzter Zugriff: 28.03.2023).

3 www.loveline.de/themen/verhuetung/; www.familienplanung.de (letzter Zugriff: 28.03.2023).

4 www.tiktok.com/@doktorsex/video/6924280480846122245 (letzter Zugriff: 28.03.2023).

5 de.wikipedia.org/wiki/Kondom (letzter Zugriff: 28.03.2023).

6 www.familienplanung.de/verhuetung/verhuetungsmethoden/kondom-fuer-ihn-sie/kondom/ (letzter Zugriff: 28.03.2023).

7 https://www.youtube.com/watch?v=o6GXDKvDHBI (letzter Zugriff: 28.03.2023).

8 https://www.tiktok.com/@lilielouiselias/video/7018167978206235910 (letzter Zugriff: 28.03.2023).

9 https://www.instagram.com/p/CRmBcAHMir3/ (letzter Zugriff: 28.03.2023).

10 https://www.instagram.com/p/CEi2NwRCqGr/ (letzter Zugriff: 28.03.2023).

11 https://www.tiktok.com/@doktorsex/video/6911242617858608386 (letzter Zugriff: 28.03.2023).

12 Nichtbinäre und trans Personen waren als Anbietende von Verhütungsbeiträgen im Sample nicht sichtbar.

13 https://www.youtube.com/watch?v=BPhtuI6t2XI (letzter Zugriff: 28.03.2023).

14 https://www.youtube.com/watch?v=8nFvUmrkjoM (letzter Zugriff: 28.03.2023).

15 https://www.youtube.com/watch?v=mlpwrKf5hm0; Minute 15:50 (letzter Zugriff: 28.03.2023).

16 https://www.tiktok.com/@tinoviral/video/7017825572738878722; Grafik im Hintergrund (letzter Zugriff: 28.03.2023).

17 35 YouTube-Videos hatten keine Kommentare bzw. die Kommentarfunktion war deaktiviert.

18 245.070 / 965 = 253,96 Kommentare.
