# Erratum zu: Verhütung auf YouTube, Instagram und TikTok

**DOI:** 10.1007/s00103-025-04174-7

**Published:** 2025-12-11

**Authors:** Nicola Döring, Stephan Lehmann, Claudia Schumann-Doermer

**Affiliations:** 1 IfMK, TU Ilmenau, Ehrenbergstraße 29, 98693 Ilmenau, Deutschland; 2 Deutsche Gesellschaft für psychosomatische Frauenheilkunde und Geburtshilfe (DGPFG), Dresden, Deutschland

**Erratum zu:**

**Bundesgesundheitsbl 2023**

10.1007/s00103-023-03698-0

In diesem Artikel hätte an den folgenden drei Stellen der Terminus ‚Twitter‘ richtigerweise ‚TikTok‘ lauten sollen:

- „Da sich die bisherige Forschung vor allem auf englischsprachiges Material bezieht und meist einzelne Methoden auf einzelnen Plattformen in den Blick nimmt, zielt die vorliegende Untersuchung darauf ab, auf der Basis eigener Vorstudien [6–8] erstmals einen breiten quantitativen Überblick über deutschsprachige Verhütungsinformationen in sozialen Medien zu geben und dabei die 3 führenden Plattformen YouTube, Instagram und Twitter vergleichend einzubeziehen.“ lautet korrekt: „Da sich die bisherige Forschung vor allem auf englischsprachiges Material bezieht und meist einzelne Methoden auf einzelnen Plattformen in den Blick nimmt, zielt die vorliegende Untersuchung darauf ab, auf der Basis eigener Vorstudien [6–8] erstmals einen breiten quantitativen Überblick über deutschsprachige Verhütungsinformationen in sozialen Medien zu geben und dabei die 3 führenden Plattformen YouTube, Instagram und TikTok vergleichend einzubeziehen.“
- „F1. Wer bietet auf YouTube, Instagram und Twitter Informationsbeiträge über Verhütung an?“ lautet korrekt: „F1. Wer bietet auf YouTube, Instagram und TikTok Informationsbeiträge über Verhütung an?“
- „Die vorliegende Inhalts- und Qualitätsanalyse beschreibt die Verhütungskommunikation auf YouTube, Instagram und Twitter.“ lautet korrekt: „Die vorliegende Inhalts- und Qualitätsanalyse beschreibt die Verhütungskommunikation auf YouTube, Instagram und TikTok.“

Der Originalbeitrag wurde korrigiert.

